# Chicago Gynecological Society, Meeting May 29th

**Published:** 1885-07

**Authors:** 

**Affiliations:** 2330 Indiana Ave.


### TRANSACTIONS OF THE CHICAGO GYNECOLOGICAL SOCIETY.

I. MARTIN. The Normal Position of the Uterus and its Relation to the Other Pelvic Organs.

*Friday evening, 29th May, 1885. Regular Meeting.*

The Society was called to order by the PRESIDENT. Dr. H. P. MERRIMAN.

I. An inaugural thesis, entitled, THE NORMAL POSITION OF THE UTERUS AND ITS RELATION TO THE OTHER PELVIC ORGANS, was presented by FRANLIN H. MARTIN, M. D., (Chicago Medical College, 1880), and read by the SECRETARY, Dr. EDWARD WARREN SAWYER.

The extreme theories of Schultze, Fritsch and Savage were opposed for the following reasons:

1. In extreme anteversion, the wave impulse would strike the posterior broad surface of the body of the uterus, and drive it down upon the bladder and anterior wall of the vagina, while, on the other hand, (the perpendicular theory of Savage), the anterior broad surface of the body would receive the impulse to an equal disadvantage, displacing the uterus backward and driving the cervix downward, while if the uterus occupied the position between these two extremes, the narrow crest of the fundus would receive the impulse in the line of the axis of the uterus and all the force would become equally distributed through all of its supports. Here, too, the organ would not so directly receive the whole impulse, as it would be equally dispersed upon its sides and the posterior ligaments and anterior supports, and its lateral attachments would receive, to an equal extent, their portion of the impulse.

2. The manner in which the bladder collapses, to our mind, precludes the possibility, or at least the probability of the uterus occupying normally the position of extreme anteversion. The bladder, when collapsed, or when empty, is a triangular shaped body,—not flat like a plate. The base corresponding to its peritoneal surface, the apex corresponding to the urethra. The posterior or inferior surface corresponds to the anterior wall of the vagina, to which it is intimately attached; the anterior wall corresponds to the symphysis, to which it is loosely attached. It is readily seen, then, that the bladder distends only in the direction of the peritoneum, or its one free surface. According to the extreme anteversion theorists, the free surface of the bladder and the uterus are in apposition. If such be the case, the uterus changes its position constantly, as the bladder normally relaxes or contracts,—this seems to us very improbable. We believe that this space is usually filled with the light coils of the small intestines.

3. The broad ligaments receive their external attachments at a point about equidistant from the center of the sacrum posteriorly, and the pubic junction anteriorly, in such a way as to divide the plane of the brim of the true pelvis into about equal halves. If the body of the uterus occupies a position in the center of the pelvis on a direct line with the ordinary attachments of these ligaments, which it is at least rational to believe is the case, it occupies a position between the perpendicular of SAVAGE, and the extreme anteversion of FRITSCH.

4. With extreme anteversion, the cervix, with the fundus occupying a position behind the symphysis would necessarily have to occupy a position far back in the pelvis, within three-fourths of one inch of the sacrum,—with a normal confirmation of parts, this is impossible without interfering with the rectum.

5. If we take the measurements of FOSTER and LITZMANN into consideration, we can at once demonstate the impracticability of the position given by SAVAGE,—*i. e.*, the perpendicular. The cervix occupies a position, normally at a distance of one and one-half inches from the sacrum, the rectum intervening. It is impossible for the uterus to assume anything like a perpendicular with the cervix in this position, on account of the anterior curve of the sacrum above, which necessitates an anterior version from the perpendicular of at least fifteen degrees.

DISCUSSION.

PROFESSOR W. W. JAGGARD was pleased with the selection of the topic, and its mode of treatment, but did not agree with Dr. MARTIN in all his conclusions.

BANDL had made a correct statement of the diversity of opinion upon this subject, in his essay on "*The Normal Position and the Normal Relationship of the Uterus, and the Pathologico-Anatomical Causes of the Symptom of Anteflexion.*" (*Archiv für Gynækologie, Band, XXVI. Heft, 3, 1884,*) read before the GYNECOLOGICAL SECTION of the *Versammlung deutscher Naturforscher und Ærzte* in Freiburg, September, 1883.

"In the course of time, almost every position of the uterus, with the exception of prolapse, has been accepted as the normal by different anatomists and gynecologists, and particularly by the more eminent ones."

KÖLLKER (1882), from a series of examinations of the cadavers of girls, from ten to eighteen years old, has concluded that the uterus is not bent, nor curved upon itself, but is straight, and that its long axis corresponds with the principal axis of the small pelvis. Its position is variable within certain limits, depending upon the condition of the bladder and rectum. This opinion coincides closely with judgments of KOHLRAUSCH (1854), LE GENDRE, (1868), FREUND, CARL BRAUN, (1857), J. MARION SIMS, (1855), LANGER, (1881). PROFESSOR PAUL F. MUNDE, in his recent excellent work on "Minor Surgical Gynecology," favors these views to the extent, that he says, "with the woman in the recumbent position, the examining finger is unable to touch the body of the uterus before or behind the cervix, if the uterus is normally situated."

BANDL, in the paper, to which allusion was made, confirms KÖLLIKER'S view. The evidence he furnishes is of a high order. His methods of investigation were:

1. The attentive examination of living women.
2. Examination and observation before and during the operation of laparotomy.
3. The bi-manual examination of the organ in cadavers, before and after abdominal section.
4. The comparative anatomical examination of many *uteri*.

Dr. PHILIP ADOLPHUS thought, with EMMET, that "there is no common standard by which to determine the proper position for the uterus in all women, but that in each individual there is a point, or plane, in the pelvis which the uterus should occupy when she is in a state of health and not pregnant." He referred in detail to EMMET'S "normal or health line," and to the pathological character of displacements above or below this line. It was a matter of relative insignificance whether or no the long axis of the uterus coincided with any particular pelvic axis.

In the concrete case, the sensations of the individual would indicate a normal or abnormal position.

PROFESSOR DANIEL T. NELSON said the uterus was fixed in a position of unstable equilibrium by the annular and other ligaments. It could move to a certain degree in every direction, and return to its original, normal position. Displacement above or below EMMET'S "health line" was productive of symptoms, if the uterus remained fixed in such a position, as was usually the case when violence caused the dislocation. Departure from the principal axis of the pelvis was a comparatively insignificant moment, viewed absolutely. The vagina and perineum are not primary supports of the uterus, and only assume this function, when, as the result of the relaxation ot the proper uterine supports, the organ is displaced downwards. This secondary character of the vaginal and perineal support was capable of demonstration by the examination of a woman in the erect attitude. Upon coughing or sneezing the uterus would descend and receive support from vagina and perineum, only to regain its original position when the excitant was removed. This remark applied exclusively to normal organs in normal position.

He wished to emphasize the statement that the rectum was not the normal receptacle for the faeces. Anatomy and physiology teach that in the normal condition the gut is empty up to the sigmoid flexure. The sigmoid flexure is a sort of valve to retain fæces.

He narrated the history of a case of retention of urine in a puerperal woman, in which the bladder was displaced towards the left iliac fossa, while the uterus was directed towards the right. He would like to ask the Fellows if this displacement, observed in a single case, corresponded with their observations.

PROFESSOR CHARLES WARRINGTON EARLE related the history of a case of retention of urine in a puerperal woman, the bladder displacing the uterus upwards and backwards. Upon the introduction of a catheter, four quarts of urine were evacuated and the pelvic viscera returned to their normal relations.

Dr. EDWARD WARREN SAWYER said the uterus had great latitude of movement antero-posteriorly and laterally; elevation above or depression below the normal plane, even to a slight degree, was productive of pain. The introduction of a pessary, which merely elevated the uterus when partially prolapsed, without altering flexion, was sufficient in many cases to afford complete relief.

While a student in the Medical Department of Harvard University, he had taken plaster casts of the vagina. Such casts were of uniform shape, while they differed in size. They were curved, convex posteriorly, concave anteriorly. They were never shaped like an S. The curve did not correspond to that of the anterior surface of the sacrum, but to the floor of the pelvis.

Dr. H. T. BYFORD thought DR. SAWYER'S experiments were faulty. When plaster of Paris was injected into the vagina, with the rectum empty, the vagina would act exactly as the rectum would under similar conditions.

THE PRESIDENT, Dr. H. P. MERRIMAN complimented the author of the paper on the careful, judicial mode of treatment of his difficult subject.

He agreed with DR. ADOLPHUS, DR. NELSON, DR. SAWYER that elevation above or depression below a certain horizontal plane was of greater importance, in the production of symptoms, than deviation from the principal pelvic axis antero-posteriorly or laterally.

The normal position of the uterus was as variable as the quantity of blood lost at a menstruation. Every woman was a law unto herself. He referred in detail to ROBERT BARNES'S theory of uterine support, and concluded by recommending BOZEMANN'S plan of columning the vagina, when a hard rubber pessary could not be borne.

PROFESSOR CHRISTIAN FENGER, M. D. (Copenhagen, 1867), and FRANKLIN H. MARTIN, M. D. (Chicago Medical College, 1880), were then elected Fellows of the Society.

W. W. JAGGARD, M. D., *Editor.*

2330 Indiana Ave., May 29, 1885.

